# Expression and Functional Evaluation of Recombinant Anti-receptor Activator of Nuclear Factor Kappa-B Ligand Monoclonal Antibody Produced in *Nicotiana benthamiana*

**DOI:** 10.3389/fpls.2021.683417

**Published:** 2021-06-23

**Authors:** Wanuttha Boonyayothin, Sirorut Sinnung, Balamurugan Shanmugaraj, Yoshito Abe, Richard Strasser, Prasit Pavasant, Waranyoo Phoolcharoen

**Affiliations:** ^1^Research Unit for Plant-Produced Pharmaceuticals, Chulalongkorn University, Bangkok, Thailand; ^2^Department of Pharmacognosy and Pharmaceutical Botany, Faculty of Pharmaceutical Sciences, Chulalongkorn University, Bangkok, Thailand; ^3^Center of Excellence in Regenerative Dentistry, Faculty of Dentistry, Chulalongkorn University, Bangkok, Thailand; ^4^Department of Pharmaceutical Sciences, School of Pharmacy at Fukuoka, International University of Health and Welfare, Okawa, Japan; ^5^Department of Applied Genetics and Cell Biology, University of Natural Resources and Life Sciences, Vienna, Austria; ^6^Department of Anatomy, Faculty of Dentistry, Chulalongkorn University, Bangkok, Thailand

**Keywords:** receptor activator of nuclear factor kappa-B ligand, plant-produced monoclonal antibody, *Nicotiana benthamiana*, transient expression, osteoclastogenesis, denosumab

## Abstract

Denosumab, an anti-receptor activator of nuclear factor-kappa B ligand antibody (anti-RANKL), is a fully human monoclonal antibody (mAb) available for the treatment of osteoporosis. In the present study, an anti-RANKL mAb was transiently expressed using the geminiviral expression system in *Nicotiana benthamiana*, and the functional activity of the plant-produced mAb was determined. The highest expression level of the plant-produced mAb was found at 8 days post-infiltration, and it was estimated to be 0.5 mg/g leaf fresh weight. The recombinant mAb from the plant crude extracts was purified by using Protein A affinity column chromatography. The plant-produced mAb demonstrated good *in vitro* affinity binding with human RANKL, as determined by RANKL-ELISA binding. The function of the plant-produced mAb was evaluated *in vitro*. CD14-positive cells isolated from human peripheral blood mononuclear cells (PBMCs) were cultured *in vitro* in the presence of human RANKL and macrophage-colony-stimulating factor (M-CSF) to stimulate osteoclastogenesis. The results demonstrated that plant-produced mAb could significantly decrease the number of osteoclasts compared to commercial denosumab. These results demonstrated that the plant-produced mAb has the potential to inhibit osteoclast differentiation and that it could be considered for osteoporosis treatment.

## Introduction

Osteoporosis is a common skeletal disease caused by an imbalance in the bone remodeling process (or bone metabolism; Lewiecki, 2010b; Cosman et al., 2014). This disease can be characterized by low bone mineral density (BMD) and microarchitectural deterioration, leading to an increase in the risk of fracture (NIH Consensus Development Panel on Osteoporosis Prevention and Therapy, 2001). Bone remodeling is an essential process that balances bone formation and bone resorption. In brief, osteoclast cells continuously resorb older and damaged bone, while osteoblast cells reconstruct new bone (Hadjidakis and Androulakis, 2006) to maintain a healthy skeleton by preventing microfractures (Sözen et al., 2017). During osteoporosis, the bone resorption rate is greater than the bone formation rate, which increases the risk of osteoporosis-related fractures.

**Figure 1:**
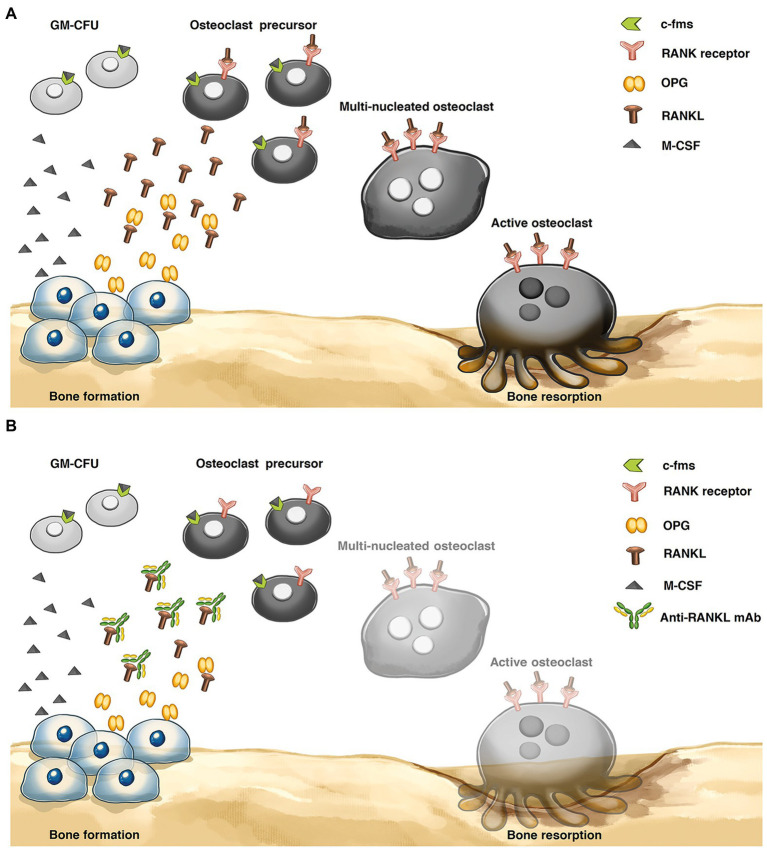
Bone remodeling process **(A)** Osteoclastic bone resorption pathway. Osteoblasts express receptor activator of nuclear factor-κB ligand (RANKL), and RANKL binds to the RANK receptor on the osteoclast precursor surface, inducing active osteoclasts. Osteoprotegerin (OPG) plays an important role in inhibiting the interaction of RANKL and RANK receptors to reduce osteoclast differentiation. **(B)** Mechanism of anti-RANKL mAb. The levels of osteoclast differentiation and activity were decreased *via* an anti-RANKL mAb, preventing the interaction between the RANKL and RANK receptors. Granulocyte-macrophage-colony-forming cell (GM-CFU), macrophage-colony-stimulating factor (M-CSF), and colony-stimulating factor-1 receptor (c-fms).

As shown in Figure 1A, the main factors regulating the mechanism of bone resorption are receptor activator of nuclear factor kappa B ligand (RANKL) and macrophage-colony-stimulating factor (M-CSF), which are presented by osteoblasts. M-CSF activates the osteoclast precursors. RANKL binds to the receptor activator of nuclear factor kappa-B (RANK) on the cell surface of osteoclast precursors to stimulate active osteoclasts and increases the bone resorption rate (Ono et al., 2020). During the bone remodeling process, osteoprotegerin (OPG) acts as an antagonist of RANKL and is provided by osteoblasts. An imbalance in the RANKL/OPG ratio can cause osteoporosis and metabolic bone diseases.

Currently, there are many treatment agents that are commonly used to decrease osteoclast differentiation, osteoclastic bone resorption and the risk of osteoporotic fracture (Lewiecki, 2010a; Zaheer et al., 2015; Tu et al., 2018). For instance, estrogen replacement therapy (ERT) is commonly used for postmenopausal osteoporosis (Wu et al., 2012). Strontium ranelate (SR) has also been reported as a drug that can reduce the risk of fracture (Clarke, 2020). Moreover, the most widely used antiresorptive agent in osteoporosis treatment is bisphosphonates, which can stimulate the apoptosis of osteoclast cells to inhibit bone resorption, but the drug is poorly absorbed and can cause gastrointestinal (GI) side effects and osteonecrosis of the jaw (ONJ) on long-term consumption (Domotor et al., 2020). Currently, one of the biological agents for osteoporosis treatment is denosumab or anti-RANKL monoclonal antibody (mAb). This mAb was approved by the US FDA for treatment of postmenopausal osteoporosis with a high fracture risk, and it can reduce the risk of spine, hip, and nonvertebral fractures (Green, 2010; Deeks, 2018). These mAbs are an alternative for patients who have upper GI problems, and they can prevent upper GI injury from bisphosphonates. Moreover, they are suitable for patients with impaired renal function (Anastasilakis et al., 2018).

Denosumab is a fully human monoclonal antibody that has an approximate molecular weight of 147 kDa. It binds with high affinity to the RANKL, similar to OPG, to prevent the interaction between RANKL and RANK and decrease the rate of bone resorption (Makras et al., 2015; Faienza et al., 2018; Figure 1B). Recently, denosumab was produced from Chinese hamster ovary (CHO) cells. Although mammalian cells show beneficial effects in recombinant therapeutic protein production, such as producing properly folded and posttranslationally modified proteins (PTMs; Khan, 2013), this process has a high production cost, a complicated technology, limited scalability, and the possibility of contaminating the product with human and animal pathogens (Yin et al., 2007; Leuzinger et al., 2013).

There are many protein expression systems available, such as mammalian cells, yeast, and bacteria, to produce recombinant therapeutic proteins. There are some drawbacks to each system. For instance, the bacterial expression system is a commonly used platform for producing recombinant proteins. The major drawback associated with the prokaryotic system is the lack of appropriate PTMs that may result in incorrect protein folding (Balamurugan et al., 2006). In contrast, mammalian cells have many beneficial aspects, but the production cost is high (Yin et al., 2007; Leuzinger et al., 2013). For yeast systems, there are many advantages, such as a rapid growth rate, the requirement for a simple growth medium, and the ability to perform PTMs. However, this system has some limitations, such as hyperglycosylation and complicated downstream processes (Gomes et al., 2016). For insect cell systems, there is a possibility of contamination with mammalian viruses, and also, proteases in the host cell might cause protein degradation (Hejnaes and Ransohoff, 2018).

To overcome these limitations, a plant-based production system is an alternative platform for recombinant therapeutic protein production. Plant expression platforms have several advantages over traditional expression platforms. For instance, plants present a lower production cost, an excellent scale-up capacity, and the lack of the chance of contaminating the product with human or animal pathogens (Pogue et al., 2010; Phoolcharoen et al., 2011; Xu et al., 2011; Chen et al., 2013; Leuzinger et al., 2013; D’aoust et al., 2017; Rattanapisit et al., 2017, 2020; Shanmugaraj et al., 2020a). Moreover, plants have the ability to perform PTMs, such as glycosylation, disulfide bond formation, phosphorylation, or proteolytic processing. PTMs play important roles in protein folding, conformational stability, and activity (Xu et al., 2011; Uversky, 2013; Zhang et al., 2017).

Because of its many advantages over other expression systems, the plant expression system could be an alternative platform that is suitable for producing recombinant proteins for therapeutic purposes. Therefore, this study aimed to transiently express anti-RANKL mAb in *Nicotiana benthamiana*. The production time of the plant-produced mAb was optimized to obtain the highest expression level. Moreover, the functional activity of the plant-produced mAb was also examined. The results indicate that the plant-produced mAb has the potential to bind with high affinity to human RANKL, and it can inhibit the differentiation and proliferation of osteoclasts.

## Materials and Methods

### Construction of a Plant Expression Vector for Producing Anti-RANKL mAb

**Figure 2:**
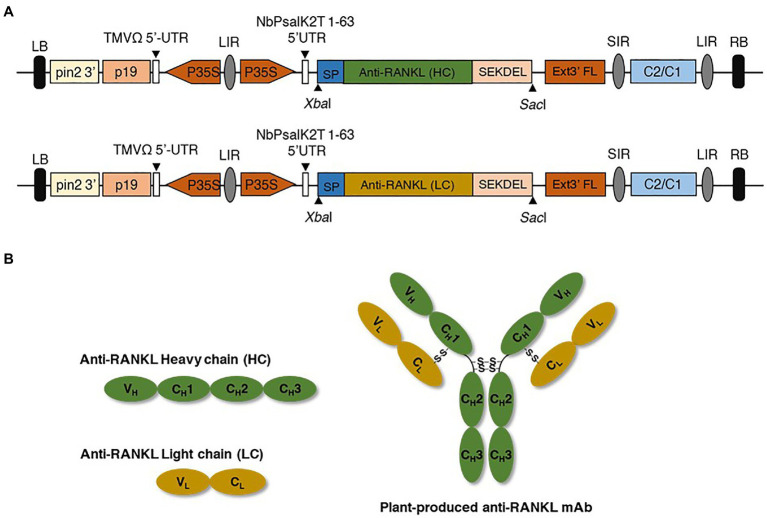
**(A)** Schematic representation of the geminiviral expression vector. P35S, Cauliflower mosaic virus (CaMV) 35S promoter; TMVΩ 5'-UTR, tobacco mosaic virus Ω 5' untranslated region; anti-RANKL mAb gene, anti-receptor activator of nuclear factor Kappa-B ligand monoclonal antibody; HC and LC, heavy and light chains of the antibody; SP, signal peptide; SEKDEL, C-terminal endoplasmic reticulum (ER) retention signal peptide; Ext3' FL, 3' full length of the tobacco extension gene; C2/C1, C1 and C2 gene of Bean Yellow Dwarf virus (BeYDV) for replication initiation protein (Rep) and RepA; p19, p19 gene from Tomato Bushy Stunt Virus (TBSV); LIR, long intergenic region of BeYDV genome; SIR, short intergenic region of BeYDV genome; LB and RB, left and right borders of the *Agrobacterium*. **(B)** Schematic and structural elements of heavy chain (HC), light chain (LC), and assembled plant-produced anti-RANKL mAb.

The amino acid sequences of the heavy chain (HC) and light chain (LC) of anti-RANKL (DrugBank accession number: DB06643; Supplementary Figure S1) were codon-optimized for *N. benthamiana*, and the gene sequences were synthesized (Bioneer, South Korea). The signal peptide (MGWSCIILFLVATATGVHS) was added to the amino terminus (N-terminus), and SEKDEL was added to the carboxyl terminus (C-terminus) of both the HC and LC. The synthesized gene was digested with *Xba*I and *Sac*I restriction enzymes (New England Biolabs, United Kingdom), and the digested products were gel-extracted and purified. In this study, a geminiviral expression vector (pBYR2eK2 Md; pBYK-2e; Chen et al., 2011) was used as an expression vector that was kindly provided by Prof. Hugh S. Mason, Arizona State University, United States. The gel-extracted product was then ligated into pBYK-2e by using T4 DNA ligase (New England Biolabs, United Kingdom), as shown in Figure 2A. The recombinant vector was further transformed into *Escherichia coli* strain DH10B by heat shock (Froger and Hall, 2007) and then transformed into *Agrobacterium tumefaciens* strain GV3101 *via* electroporation. *Agrobacterium* clones were confirmed by PCR using gene-specific forward and reverse primers. The PCR cycling conditions were as follows: an initial denaturation at 94°C for 2 min, followed by 35 cycles of 94°C for 30 s, 55°C for 30 s, and 72°C for 30–60 s, and a final extension at 72°C for 5 min. Taq DNA polymerase (Vivantis Technologies, Malaysia) was used for amplification, and the PCR products were separated on 1% agarose gel. Positive *Agrobacterium* clones were used for further experiments.

### Transient Expression of Anti-RANKL mAb in *N. benthamiana*

*Agrobacterium tumefaciens* harboring the plant expression vector containing either HC or LC was cultured on selective Luria–Bertani (LB) medium containing 50 μg/ml rifampicin, gentamicin, and kanamycin and then incubated at 28°C overnight with continuous shaking at 200 rpm. After incubation, the bacterial suspension was centrifuged at 4,000 *g* for 10 min, and the pellet was resuspended in infiltration buffer [10 mM 2-N-morpholino-ethanesulfonic acid (MES) and 10 mM MgSO4, pH 5.5] to obtain a final optical density (OD_600_) of 0.4. Then, an *Agrobacterium* suspension containing HC and LC was mixed equally and co-infiltrated into 6- to 8-week-old wild-type *N. benthamiana* by syringe infiltration and incubated at 28°C with a 16-h light/8-h dark cycle. The infiltrated leaves were harvested at 2, 4, 6, 8, and 10 days post-infiltration (dpi), and ELISA was performed for protein quantification. The large-scale production of mAb was performed by using vacuum infiltration.

### Anti-RANKL mAb Extraction and Expression-Level Quantification

The infiltrated leaves were harvested at the appropriate dpi and extracted with 1x phosphate-buffered saline (PBS; 137 mM NaCl, 2.68 mM KCl, 10.1 mM Na_2_HPO_4_, and 1.76 mM KH_2_PO_4_, pH 7.4). The plant crude extract was centrifuged at 26,000 *g* for 40 min at 4°C to remove the cell debris. Sandwich ELISA was performed to quantify the plant-produced mAb expression level. Briefly, 96-well ELISA plates (Greiner Bio One GmbH, Austria) were coated with 50 μl anti-human IgG-Fc fragment-specific (Abcam, United Kingdom) prepared at 1:1,000 in 1x PBS (pH 7.4) and incubated overnight. Then, the plates were washed with 1x PBST (1xPBS containing 0.05% Tween-20) three times and blocked with 200 μl 5% skim milk in 1x PBS. After washing three times with 1x PBST, the plate was coated with commercial denosumab (AMGEN, United States) or plant-produced mAb at varying dilutions in 1x PBS and incubated at 37°C for 2 h. Each sample was loaded in triplicate wells of an ELISA plate. Then, the plate was washed three times with 1x PBST followed by HRP-conjugated anti-human kappa antibody (Southern Biotech, United States) with a dilution of 1:1,000 in 1x PBS and incubated for 1 h at 37°C. Then, the plate was washed three times with 1x PBST and developed by adding SureBlue™ TMB 1-Component Microwell Peroxidase Substrate (Promega, United States). To stop the reaction, 1 M H_2_SO_4_ was added, and the absorbance was measured using a microplate reader at an optical density of 450 nm (OD_450_).

### Purification of Anti-RANKL mAb

The crude extract from the infiltrated leaves was clarified by centrifugation, and the supernatant was filtered through a 0.45-micron filter (Millipore Sigma, United States) before loading into a Protein A affinity chromatography column. Amintra® Protein A Resin (Expedeon, United Kingdom) was packed into the column and equilibrated with 1x PBS (pH 7.4). After equilibration, the filtered crude extract was loaded on the column, and the column was further washed with 1x PBS (pH 7.4). The protein was eluted with elution buffer (0.1 M glycine, pH 2.7) and neutralized with 1.5 M Tris-HCl (pH 8.8). Then, the purified protein was separated by sodium dodecyl sulfate–polyacrylamide gel electrophoresis (SDS–PAGE), followed by either staining with Coomassie Brilliant Blue (AppliChem, Germany) or carrying out western blotting.

### SDS–PAGE and Western Blotting

Sodium dodecyl sulfate–polyacrylamide gel electrophoresis and western blotting were performed to determine the purity of the plant-produced mAb. Commercial denosumab (AMGEN, United States) was used as a positive control. The samples were analyzed under reducing and nonreducing conditions. For nonreducing conditions, loading buffer [125 mM Tris-HCl pH 6.8, 12% (w/v) SDS, 10% (v/v) glycerol, and 0.001% (w/v) bromophenol blue] was added, and the sample was denatured at 95°C for 5 min. The denatured samples were separated on 6–10% polyacrylamide gels. For reducing conditions, the sample was mixed with a loading buffer containing 22% β-mercaptoethanol, denatured at 95°C for 5 min, and then separated on 15% polyacrylamide gels. The polyacrylamide gels were stained with Coomassie Brilliant Blue (AppliChem, Germany), and the bands were visualized. For western blot analysis, the separated protein in the polyacrylamide gel was transferred to a nitrocellulose membrane (Bio-Rad, United States). The membrane was blocked with 5% skim milk in 1x PBS (pH 7.4) and then incubated either with HRP-conjugated anti-human gamma antibody (The Binding Site, United Kingdom) or with HRP-conjugated anti-human kappa antibody (Southern Biotech, United States) at a 1:5,000 dilution in 3% skim milk prepared in 1x PBS (pH 7.4). After incubation, the membrane was washed three times with 1x PBST, and the membrane was developed with Amersham ECL prime western blotting detection reagent (GE Healthcare, United Kingdom).

### N-Glycan Analysis of the Plant-Produced mAb

The purified plant-produced mAb was separated on 15% polyacrylamide gels under reducing conditions and stained with Coomassie Brilliant Blue (AppliChem, Germany), and the bands were visualized. The HC was excised from the gel, S-alkylated, and digested with trypsin. The tryptic glycopeptides were analyzed by using liquid chromatography–electrospray ionization–mass spectrometry (LC–ESI–MS) as described previously in a study by Strasser et al. (2008).

### Antibody Structure Characterization

Structure characterizations were performed using CD and NMR spectroscopic techniques. For CD spectroscopy, plant-produced mAb (10 μM) was dissolved in PBS buffer (pH 7.4). The CD spectra were recorded at room temperature using a quartz cell with a 1 mm optical path length on a J-720 W CD spectropolarimeter (JASCO, Tokyo, Japan). For NMR spectroscopy, NMR samples (100 μM) of plant-produced mAb were dissolved in a PBS buffer (pH 7.4) containing 10% v/v D_2_O. NMR spectra were recorded on a Varian Unity INOVA 600 spectrometer (Varian, Palo Alto, CA, United States).

### Binding Efficiency of Plant-Produced mAb to Human RANKL Protein

To investigate the binding activity of plant-produced mAb, a 96-well ELISA plate (Greiner Bio One GmbH, Austria) was coated with 3 μg/ml human RANKL protein (ProSpec-Tany TechnoGene Ltd., Israel) and incubated at 37°C for 4 h. Then, the plate was blocked with 200 μl of 5% skim milk (BD, Franklin Lakes, NJ, United States) in 1x PBS at 37°C for 2 h and washed three times with 1x PBST. Serial dilutions of plant-produced mAb, commercial denosumab (as a positive control), and human IgG1 kappa isotype antibody (Abcam, United Kingdom) were added to triplicate wells and incubated at 37°C for 2 h. After washing three times with 1x PBST, the plate was coated with HRP-conjugated anti-human gamma antibody (The Binding Site, United Kingdom) in 1x PBS at 1:1,000 and incubated at 37°C for 1 h. Then, the plate was developed by adding SureBlue™ TMB 1-Component Microwell Peroxidase Substrate (Promega, the United States), and the reaction was stopped by 1 M H_2_SO_4_. Then, the absorbance was measured using a microplate reader at an optical density of 450 nm (OD_450_).

### Functional Evaluation of the Plant-Produced mAb

#### Isolation of CD14^+^ Monocytes

Human peripheral blood mononuclear cells (PBMCs) were isolated from buffy coats obtained from The Thai Red Cross Institute. Briefly, the buffy coats were diluted with equal amounts of Dulbecco’s phosphate-buffered saline (DPBS) containing 2% fetal bovine serum (FBS; Thermo Fisher Scientific, United States). The diluted buffy coats were gently overlaid onto Ficoll-Paque PLUS (GE Healthcare, United Kingdom) and centrifuged at 400 *g* for 30 min without braking. After centrifugation, PBMCs at the interface between the plasma and the Ficoll-Paque PLUS were collected and washed with ice-cold DPBS containing 2% FBS. CD14^+^ monocytes were isolated from the PBMCs by incubation with MACS CD14^+^ microbeads (Miltenyi Biotec, Germany) for 15 min at 4°C. The cells were washed with buffer (DPBS containing 0.5% BSA and 2 mM EDTA) and passed through a MACS cell separator. The CD14^+^ monocytes were collected for further experiments.

#### Induction of Osteoclast and Inhibition

To induce osteoclasts, CD14^+^ monocytes were seeded at a density of 1 × 10^6^ cells/well in 24-well plates and cultured in 1 ml of α-minimal essential medium (α-MEM; Thermo Fisher Scientific, United States) containing 50 ng/ml M-CSF and 100 ng/ml human RANKL protein (ProSpec-Tany TechnoGene Ltd., Israel). The inhibitory experiments were performed by the addition of either plant-produced mAb or commercial denosumab (AMGEN, United States) to obtain a final concentration of 500 ng/ml. The anti-SARS-CoV mAb CR3022 (500 ng/ml; Rattanapisit et al., 2020) was used as a control. Fifty percent of the medium was replaced every 3 days with fresh medium containing 50 ng/ml M-CSF, 100 ng/ml human RANKL protein, and 500 ng/ml each of plant-produced mAb, denosumab, or CR3022 mAb, and the cultures were then maintained for 15 days. The experiment was performed in triplicate.

#### Tartrate-Resistant Acid Phosphatase Staining

Tartrate-resistant acid phosphatase staining (TRAP) was carried out using a commercial kit (Takara Bio, Japan). TRAP-positive multinucleated cells containing more than three nuclei were identified as osteoclasts and were counted under a microscope. Four fields were randomly selected, and pictures were taken by using Axio Observer Z1 and ZEN pro (ZEISS International, Oberkochen, Germany).

#### Statistical Analysis

The data are presented as the mean ± SEM. To assess the statistical significance of the differences, an unpaired two-sample t-test was performed, with values of *p* ≤ 0.0001 considered statistically significant.

## Results

### Transient Expression of Anti-RANKL mAb in *N. benthamiana* Leaves

**Figure 3:**
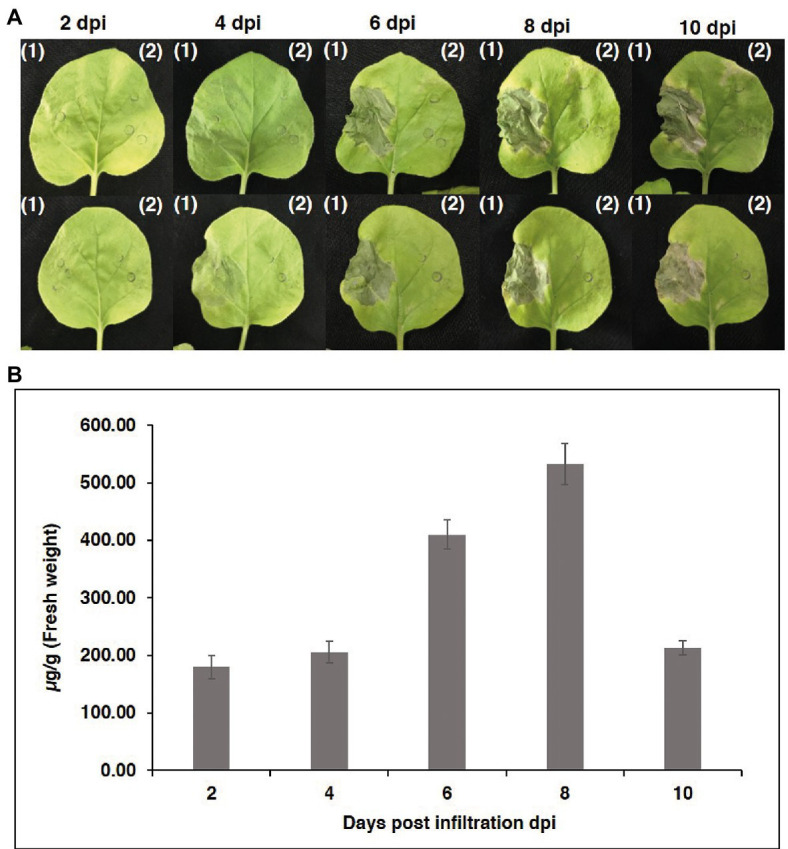
Transient expression of anti-RANKL mAb in *Nicotiana benthamiana* leaves. **(A)** Phenotype of leaves on days 2, 4, 6, 8, and 10 after agroinfiltration. (1) *N. benthamiana* leaves were co-infiltrated with *Agrobacterium tumefaciens* harboring pBYK-2e-anti-RANKL HC + LC and (2) *A. tumefaciens* without an expression vector. **(B)** Infiltrated leaves were harvested on days 2, 4, 6, 8, and 10 from three individual plants each day post-infiltration and quantified by ELISA. The data are shown as the mean ± standard deviation (SD) of triplicate.

To produce an anti-RANKL mAb, the nucleotide sequences of the HC and LC were modified with *N. benthamiana*-optimized codons. The genes were cloned into a geminiviral expression vector (pBYR2eK2 Md; pBYK-2e) using *Xba*I and *Sac*I restriction sites and subsequently electroporated into *A. tumefaciens* strain GV3101. Wild-type *N. benthamiana* leaves were co-infiltrated with *A. tumefaciens* harboring pBYK-2e-anti-RANKL HC and LC to produce assembled antibodies, including 2HC and 2 LC (Figure 2B). After agroinfiltration, strong leaf necrosis was observed, which was related to days post-infiltration (dpi; Figure 3A). The plant-produced mAb was expressed at the highest level at 8 dpi, up to 0.5 mg/g leaf fresh weight (Figure 3B).

### Purification of Anti-RANKL mAb From *N. benthamiana* Leaves

**Figure 4:**
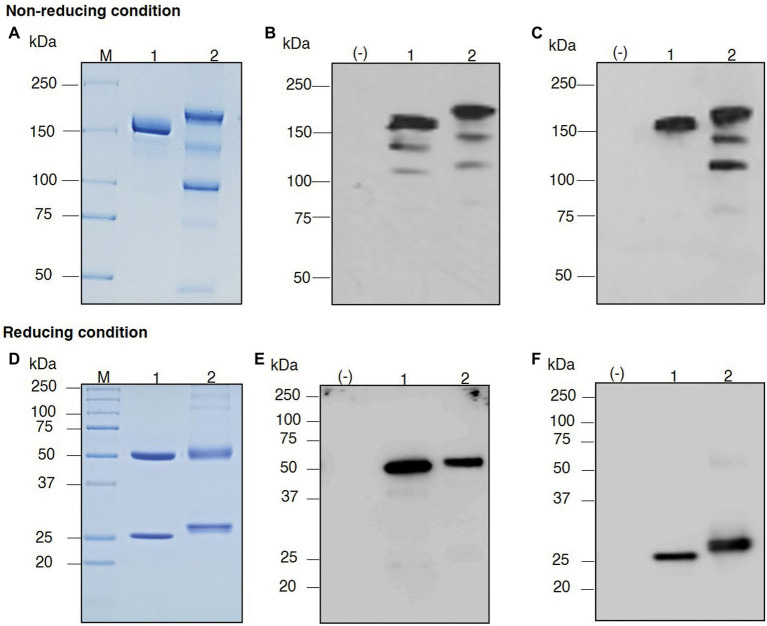
Sodium dodecyl sulfate–polyacrylamide gel electrophoresis (SDS–PAGE) of purified plant-produced anti-RANKL mAb under nonreducing conditions and reducing conditions. Crude extracts from the infiltrated leaves were purified by a Protein A affinity chromatography column. The purified product was analyzed by SDS–PAGE staining with Coomassie Blue **(A,D)**. For western blot analysis, the separated proteins in the polyacrylamide gel were transferred to a nitrocellulose membrane, and the membrane was detected with either HRP-conjugated anti-human gamma chain antibody **(B,E)** or anti-human kappa chain antibody **(C,F)**. Lane M, Protein ladder; Lane (−), Wild-type crude extract; Lane 1, Commercial denosumab; Lane 2, Purified plant-produced anti-RANKL mAb.

The plants were vacuum-infiltrated, and then the infiltrated leaves were harvested at the appropriate time point. The plant-produced mAb was purified from the plant crude extract using Protein A affinity column chromatography. SDS–PAGE and western blotting were performed to verify the purified plant-produced mAb. The SDS–PAGE gel was stained with Coomassie Brilliant Blue stain to visualize the separated protein bands. Western blotting was performed with anti-human gamma-HRP and anti-human kappa-HRP antibodies. For nonreducing conditions, the assembled antibody was confirmed to be in a tetrameric form with a molecular size of approximately 150 kDa, similar to the commercial denosumab (Figures 4A–C, Lanes 1 and 2).

Thus, the HC and LC of anti-RANKL mAb can be produced in *N. benthamiana* leaves. In reducing conditions, the protein bands were detected at molecular sizes of approximately 50 and 25 kDa, which correspond to the HC and LC of the antibody, similar to the commercial denosumab (Figures 4D–F, Lanes 1 and 2). No protein band was observed in the wild-type control leaf extract as expected [Figures 4A–F, Lane (−)]. These results indicated that the co-infiltration of genes encoding HC and LC produced the assembled anti-RANKL mAb in *N. benthamiana* leaves.

### N-Glycan Analysis of Plant-Produced mAb

**Figure 5:**
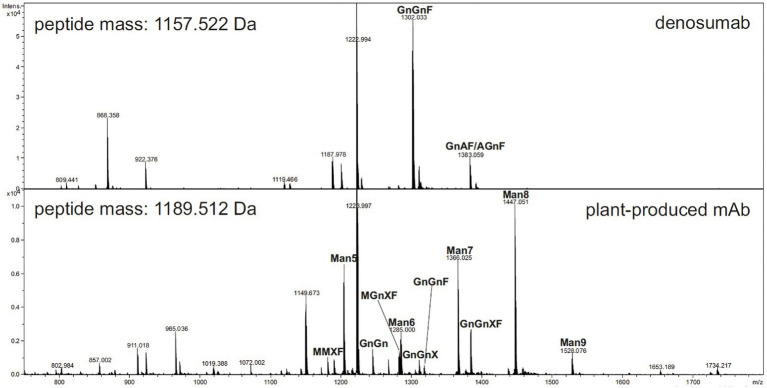
Liquid chromatography–electrospray ionization–mass spectrometry (LC–ESI–MS) of tryptic glycopeptides from anti-RANKL monoclonal antibody (mAb) produced in wild-type *N. benthamiana* plants (see www.proglycan.com for a detailed explanation of the glycan structure abbreviations.)

Liquid chromatography–electrospray ionization–mass spectrometry was performed for the analysis of the glycopeptides from the plant-produced mAb. The results indicated that the mAb displays mainly oligomannosidic N-glycans (Man5-Man9) and small amounts of complex-type and truncated N-glycans (e.g., GnGnXF), as shown in Figure 5.

### Antibody Structure Characterization

**Figure 6:**
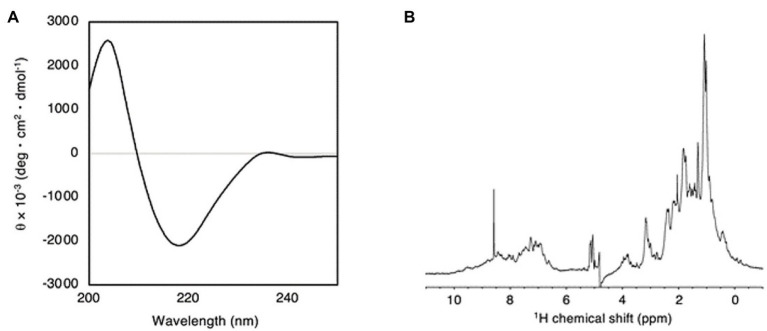
**(A)** Far-UV CD spectrum of plant-produced anti-RANKL mAb at 25°C. **(B)** ^1^H NMR spectrum of plant-produced anti-RANKL mAb at pH 7.5 and 25°C.

The secondary and tertiary structures of the plant-produced mAb were examined by using CD and NMR spectroscopy techniques. CD spectrum analysis indicated a negative absorbance at 218 nm, which represents the typical β-sheet structure. In ^1^H-NMR spectroscopy, since the signals of up-field methyl and dispersed aromatic protons were observed, the tertiary structures were retained. Consequently, the plant-produced mAb has a β-sheet-rich structure similar to the immunoglobulin-fold (Figure 6).

### Binding Properties of Plant-Produced mAb to Human RANKL

**Figure 7:**
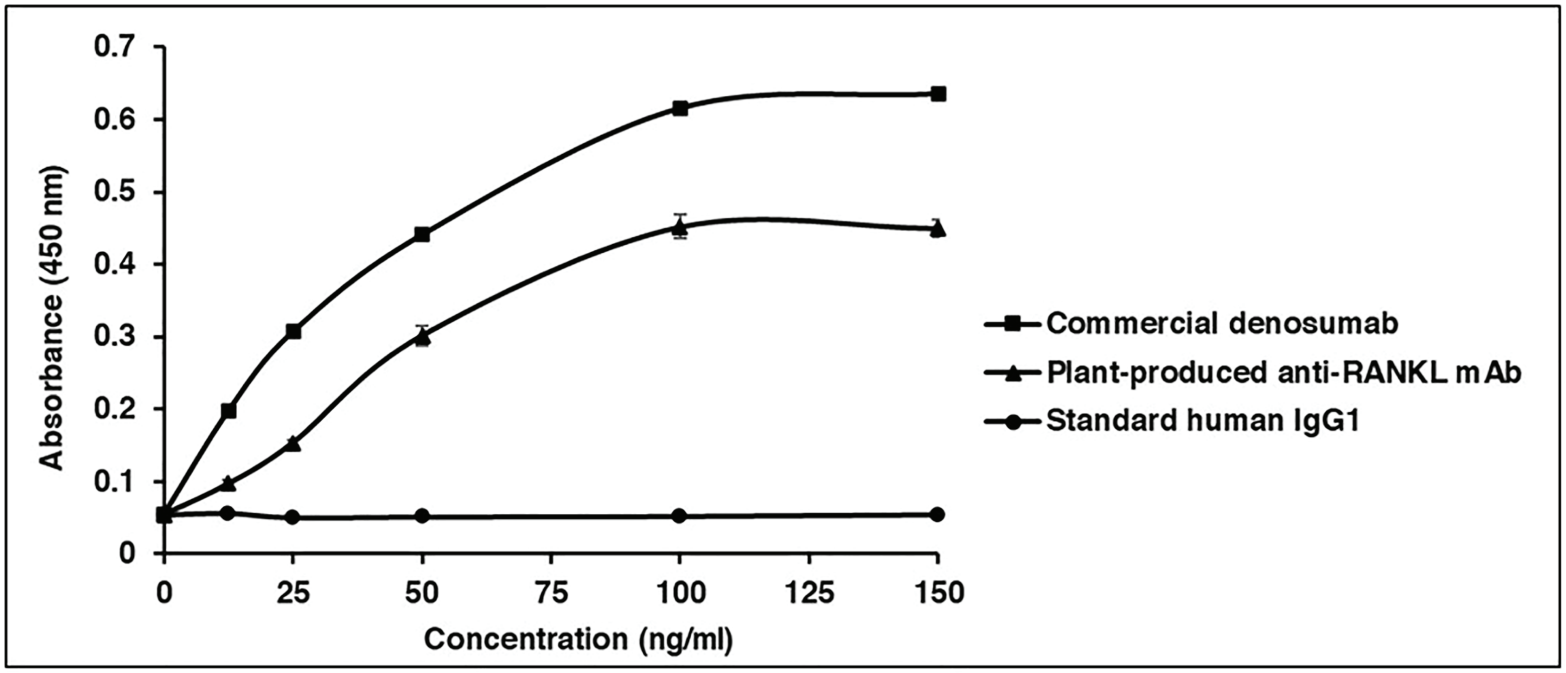
Binding properties of plant-produced anti-RANKL mAb to human RANKL. ELISA was used to investigate the specific binding. The purified plant-produced mAb, standard human immunoglobulin G (IgG)1 (as a negative control), and commercial denosumab (as a positive control) were added to the plates coated with commercial human RANKL. The binding activity of antibody was detected with HRP-conjugated anti-human gamma chain antibody. The data are shown as the mean ± SD of triplicates.

Receptor Activator of Nuclear Factor Kappa-B Ligand (RANKL)-ELISA binding analysis was performed to examine the specific binding between the plant-produced mAb and human RANKL. Serial dilutions of purified plant-produced mAb, standard human IgG1 (as a negative control), and commercial denosumab (as a positive control) were incubated with captured human RANKL in a 96-well plate. Commercial denosumab and plant-produced mAb presented signals when detected with anti-human gamma-HRP. These results demonstrated that both the commercial denosumab and the plant-produced mAb had the potential to bind to human RANKL, while human IgG1 did not show binding properties (Figure 7).

### *In vitro* Functional Analysis

**Figure 8:**
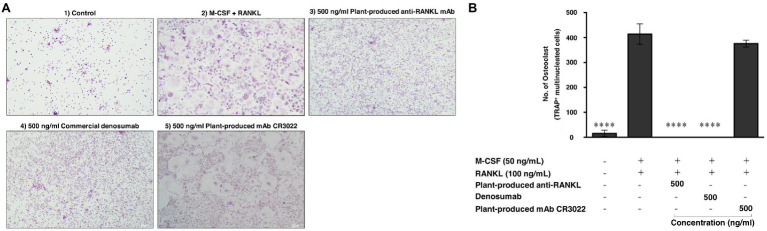
Plant-produced anti-RANKL mAb suppresses osteoclast differentiation. CD14^+^ monocytes were cultured under different conditions for 15 days following (1) control, (2) M-CSF and RANKL, (3) 500 ng/ml plant-produced anti-RANKL mAb, (4) 500 ng/ml commercial denosumab, and (5) 500 ng/ml plant-produced mAb CR3022. **(A)** Representative images of TRAP staining. **(B)** Quantification of TRAP^+^ osteoclasts is shown as the mean and SEM of triplicate ^∗∗∗∗^*p* ≤ 0.0001.

To examine the effect of plant-produced mAb on osteoclastogenesis, CD14^+^ monocytes were cultured with essential factors, including M-CSF (50 ng/ml) and human RANKL protein (ProSpec-Tany TechnoGene Ltd., Israel; 100 ng/ml). As shown in Figure 8A, CD14^+^ cells differentiated into osteoclasts as judged by their multinucleated appearance and positive staining of TRAP. The addition of both plant-produced mAb and commercial denosumab significantly reduced osteoclast differentiation at a dose of 500 ng/ml (*p* ≤ 0.0001) compared with the control condition of adding M-CSF and RANKL. Comparable effects of both antibodies were found. In contrast, the addition of the plant-produced mAb CR3022 did not affect osteoclast formation (Figure 8B).

## Discussion

Currently, the majority of therapeutic proteins, including vaccines, antibodies, and biologics, are produced from mammalian and microbial platforms. However, there are some drawbacks of these technology platforms, such as a lack of PTMs, high production cost, and scalability limitations. Recently, plants have been considered as bioreactors that can overcome this problem because they can efficiently produce large volumes of recombinant proteins, can perform PTMs, and can increase production cost-effectiveness (Uversky, 2013; Tusé et al., 2014; Chen and Davis, 2016; Chen, 2018; Shanmugaraj and Phoolcharoen, 2021).

There are many plant-produced proteins that are at different stages in clinical trials, and a few have been approved by the FDA. For instance, alpha-galactosidase-A (moss-aGal) has been used for Fabry disease (phase 1; Veen et al., 2020), HIV-neutralizing human mAb 2G12 (phase 1; Tremouillaux-Guiller et al., 2020), HA VLP influenza vaccine (phase 2; Pillet et al., 2019), anti-Ebola IgG cocktail (ZMApp) for the treatment of Ebola infection (phase 2/3; Davey et al., 2016) and glucocerebrosidase enzyme (ELELYSO) for Gaucher’s disease (Fox, 2012). *N. benthamiana* is the preferred plant system for recombinant protein expression. This plant is genetically well known and easily manipulated, is a nonfood/nonfeed crop, and produces high biomass (Xu et al., 2012).

In this study, an anti-RANKL mAb was produced in *N. benthamiana* and its biological activity was investigated. The results showed that the mAb can be transiently expressed in *N. benthamiana* by using a geminiviral replicon vector. The geminiviral expression system was modified from the genome structure of the bean yellow dwarf virus to improve the protein expression level (Chen et al., 2011). Previous studies have shown that many recombinant proteins can be transiently expressed from geminiviral replicon systems. For instance, enterotoxin B and the Ebola immune complex (EIC; including additional antigens or mAbs produced by using a geminiviral vector) were successfully expressed in *N. benthamiana* by using a geminiviral vector (Hefferon and Fan, 2004; Phoolcharoen et al., 2011). Rattanapisit et al. (2017) reported that the geminiviral expression system could be used to produce human osteopontin and showed the potential of recombinant osteopontin to induce periodontal ligament differentiation. Recently, the receptor-binding domain (RBD) of SARS-CoV-2 and the anti-SARS-CoV mAbs, CR3022, H4, and B38, were rapidly produced in *N. benthamiana* during the COVID-19 pandemic (Rattanapisit et al., 2020; Shanmugaraj et al., 2020b).

The nucleotide sequences of the anti-RANKL mAb were optimized with *N. benthamiana* codon usage to enhance recombinant protein expression, and the Ser-Glu-Lys-Asp-Glu-Leu (SEKDEL) sequences were added to the C-terminus to retain the plant-produced mAb in the endoplasmic reticulum (ER). The ER could ensure its proper folding and the assembly structure of antibodies with correct disulfide bond formation and glycosylation (Kleizen and Braakman, 2004; Braakman and Bulleid, 2011; Aebi, 2013). Furthermore, the expression level of the antibody was improved by adding SEKDEL to the amino acid sequence (Petruccelli et al., 2006) because the ER compartment is appropriate for protein retention due to a lack of proteases that can degrade the recombinant protein.

As shown in the results, the plant-produced mAb was expressed with the highest expression level of up to 0.5 mg/g fresh weight at 8 dpi (Figure 3B). Previously, some studies demonstrated that the production time of recombinant protein in plants was different and dependent on many factors, including the protein expression system and the specific protein molecules. Sheludko et al. (2007) indicated that the maximum rGFP expression level in plants obtained from the CaMV 35S promoter system with co-expression of TBSV P19 at 3 dpi remained constant until 8 dpi. Furthermore, transient expression of the recombinant EIC in *N. benthamiana* using a geminiviral vector was found to attain a maximum level at 4 dpi (Phoolcharoen et al., 2011).

In this study, N-glycan analysis of the mAb produced from wild-type *N. benthamiana* showed that it mainly had oligomannosidic N-glycans and minor amounts of complex-type N-glycans (Figure 5). The oligomannosidic structures are derived from limited processing of the N-glycans due to the retention of the mAb in the ER. The minor amounts of processed complex N-glycans are likely caused by incomplete retention and overload of the KDEL-mediated Golgi-to-ER retrieval pathway. The commercial denosumab produced in CHO cells displays complex N-glycans with a core α1,6-fucose that are typically found on secreted recombinant mAbs produced in mammalian cells. The N-glycan processing pathway in the Golgi apparatus differs between plants and mammals, which results in the presence of different N-glycan modifications on secreted recombinant glycoproteins (Gomord et al., 2004; Saint-Jore-Dupas et al., 2007; Schähs et al., 2007; Strasser, 2016; Montero-Morales and Steinkellner, 2018; Schoberer and Strasser, 2018). β1,2-Xylose of the conserved core complex N-glycan is not present in mammalian N-glycans. In addition, plant N-glycans also attach plant-specific α1,3-fucose residues to the complex core N-glycan instead of the core α1,6-fucose found in mammals. It is well established that the absence of the core α1,6-fucose reduces the affinity of the Fc domain of mAbs to bind to distinct Fc receptors that modulate the immune response (Wang and Ravetch, 2019). According to Stelter et al. (2020), a recombinant mAb with plant-specific N-glycans displayed reduced binding to Fcγ receptors, indicating that the plant-specific core α1,3-fucose has a similar effect. To prevent the attachment of these plant-specific complex N-glycan modifications, the plant-produced anti-RANKL mAb was designed to be retained in the ER.

In this study, we did not investigate the role of different N-glycans in the function of plant-produced anti-RANKL mAb. However, the proof-of-concept study shows that the plant-produced anti-RANKL mAb with primarily oligomannosidic N-glycans has comparable bioactivity to the commercial denosumab produced in a mammalian cell. In future studies, the role of different N-glycan modifications on osteoclast inhibition will be investigated using well-established glycoengineering approaches (Montero-Morales and Steinkellner, 2018; Schoberer and Strasser, 2018; Stelter et al., 2020).

A previous study showed that commercial denosumab provided the ability to bind specifically to human RANKL but not murine RANKL, human TRAIL, or other human TNF family members. In this study, a RANKL-ELISA binding assay, conducted according to Kostenuik et al. (2009), was performed to investigate the *in vitro* binding of the plant-produced mAb in comparison with commercial denosumab. The results showed that plant-produced anti-RANKL mAb demonstrated good binding potential to human RANKL, while the commercial antibody presented a higher binding signal and the standard human IgG1 (as a negative control) did not show any binding signal (Figure 7). As shown in Supplementary Figure S1, the anti-RANKL mAb amino acid sequences obtained from DrugBank (accession number: DB06643) were different from the commercialized antibody in the patent (Robblee et al., 2017). Therefore, the difference in amino acid sequences might have an impact on its binding affinity.

The bioactivity of plant-produced mAb on osteoclast inhibition was investigated. Human PBMCs were isolated and induced to differentiate into osteoclasts by M-CSF and RANKL. The cultures were treated with each plant-produced mAb, commercial denosumab, and mAb CR3022 (negative control). Our results indicated that plant-produced mAb and commercial denosumab both had the potential to inhibit osteoclast differentiation at the same concentration when tested on the CD14^+^ cells *in vitro*, whereas no inhibition potential was shown with mAb CR3022 (Figures 8A,B). This result was consistent with an earlier study that demonstrated that commercial denosumab could inhibit osteoclast differentiation *in vitro* at concentrations up to 3 nM (approximately 500 ng/ml; Kostenuik et al., 2009).

In summary, the results showed that plant-based expression systems can rapidly produce an assembled anti-RANKL mAb in *N. benthamiana*. The optimal production period was 8 dpi, with the highest expression level reaching 0.5 mg/g leaf fresh weight. There was good binding efficacy between plant-produced mAb and human RANKL. The functional *in vitro* study demonstrated that the plant-produced mAb reduced osteoclast differentiation. Therefore, these results may support the concept of a cost-effective and rapid production platform for anti-RANKL mAb and other biopharmaceutical products. However, an *in vivo* study should be performed to further confirm the efficacy and safety of the plant-produced mAb.

## Data Availability Statement

The original contributions presented in the study are included in the article/supplementary material, further inquiries can be directed to the corresponding author.

## Ethics Statement

The studies involving human participants were reviewed and approved by the human subject ethics board of the Faculty of Dentistry, Chulalongkorn University. The patients/participants provided their written informed consent to participate in this study.

## Author Contributions

WP and PP designed all of the experiments. WB, SS, BS, YA and RS performed all of the experiments. All authors analyzed the data and contributed to the manuscript preparation.

### Conflict of Interest

The authors declare that the research was conducted in the absence of any commercial or financial relationships that could be construed as a potential conflict of interest.

## References

[ref1] Aebi M. (2013). N-linked protein glycosylation in the ER. Biochim. Biophys. Acta Mol. Cell Res. 1833, 2430–2437. 10.1016/j.bbamcr.2013.04.001, PMID: 23583305

[ref2] Anastasilakis A. D. Polyzos S. A. Makras P. (2018). THERAPY OF ENDOCRINE DISEASE: Denosumab vs bisphosphonates for the treatment of postmenopausal osteoporosis. Eur. J. Endocrinol. 179, R31–R45. 10.1530/EJE-18-0056, PMID: 29691303

[ref3] Balamurugan V. Sen A. Saravanan P. Singh R. K. (2006). Biotechnology in the production of recombinant vaccine or antigen for animal health. J. Anim. Vet. Adv. 5, 487–495.

[ref4] Braakman I. Bulleid N. J. (2011). Protein folding and modification in the mammalian endoplasmic reticulum. Annu. Rev. Biochem. 80, 71–99. 10.1146/annurev-biochem-062209-093836, PMID: 21495850

[ref5] Chen Q. (2018). “Chapter seven - recombinant therapeutic molecules produced in plants,” in Advances in Botanical Research. ed. Kuntz M. (United States: Academic Press Inc), 207–244.

[ref6] Chen Q. Davis K. R. (2016). The potential of plants as a system for the development and production of human biologics. F1000Res. 5:912. 10.12688/f1000research.8010.1, PMID: 27274814 PMC4876878

[ref7] Chen Q. He J. Phoolcharoen W. Mason H. S. (2011). Geminiviral vectors based on bean yellow dwarf virus for production of vaccine antigens and monoclonal antibodies in plants. Hum. Vaccin. 7, 331–338. 10.4161/hv.7.3.14262, PMID: 21358270 PMC3166492

[ref8] Chen Q. Lai H. Hurtado J. Stahnke J. Leuzinger K. Dent M. (2013). Agroinfiltration as an effective and scalable strategy of gene delivery for production of pharmaceutical proteins. Adv. Tech. Biol. Med. 1:103. 10.4172/atbm.1000103, PMID: 25077181 PMC4113218

[ref9] Clarke B. L. (2020). “Strontium,” in Encyclopedia of Bone Biology. ed. M. Zaidi, 652–665.

[ref10] Cosman F. De Beur S. J. Leboff M. S. Lewiecki E. M. Tanner B. Randall S.. (2014). Clinician’s guide to prevention and treatment of Osteoporosis. Osteoporos. Int. 25, 2359–2381. 10.1007/s00198-014-2794-2, PMID: 25182228 PMC4176573

[ref11] D’aoust M.-A. Couture M. Ors F. Trepanier S. Lavoie P.-O. Dargis M. (2017). Recombinant Influenza Virus-Like Particles (VLPs) Produced in Transgenic Plants Expressing Hemagglutinin. United States patent application.

[ref12] Davey R. T. Jr. Dodd L. Proschan M. A. Neaton J. Neuhaus Nordwall J. Koopmeiners J. S.. (2016). A randomized, controlled trial of ZMapp for Ebola virus infection. N. Engl. J. Med. 375, 1448–1456. 10.1056/NEJMoa1604330, PMID: 27732819 PMC5086427

[ref13] Deeks E. D. (2018). Denosumab: a review in postmenopausal Osteoporosis. Drugs Aging 35, 163–173. 10.1007/s40266-018-0525-7, PMID: 29435849

[ref14] Domotor Z. R. Vorhendi N. Hanak L. Hegyi P. Kiss S. Csiki E.. (2020). Oral treatment with bisphosphonates of Osteoporosis does not increase the risk of severe gastrointestinal side effects: a meta-analysis of randomized controlled trials. Front. Endocrinol. 11:573976. 10.3389/fendo.2020.573976, PMID: 33240217 PMC7683730

[ref15] Faienza M. F. Chiarito M. D’amato G. Colaianni G. Colucci S. Grano M.. (2018). Monoclonal antibodies for treating osteoporosis. Expert. Opin. Biol. Ther. 18, 149–157. 10.1080/14712598.2018.1401607, PMID: 29113523

[ref16] Fox J. L. (2012). First plant-made biologic approved. Nat. Biotechnol. 30, 472–472. 10.1038/nbt0612-472

[ref17] Froger A. Hall J. E. (2007). Transformation of plasmid DNA into *E. coli* using the heat shock method. J. Vis. Exp. 6:253. 10.3791/253, PMID: 18997900 PMC2557105

[ref18] Gomes A. Byregowda S. Veeregowda B. Vinayagamurthy B. (2016). An overview of heterologous expression host systems for the production of recombinant proteins. Adv. Anim. Vet. Sci. 4, 346–356. 10.14737/journal.aavs/2016/4.7.346.356

[ref19] Gomord V. Sourrouille C. Fitchette A. C. Bardor M. Pagny S. Lerouge P.. (2004). Production and glycosylation of plant-made pharmaceuticals: the antibodies as a challenge. Plant Biotechnol. J. 2, 83–100. 10.1111/j.1467-7652.2004.00062.x, PMID: 17147602

[ref20] Green W. (2010). Denosumab (Prolia) injection: a new approach to the treatment of women with postmenopausal Osteoporosis. Pharm. Ther. 35, 553–559.

[ref21] Hadjidakis D. J. Androulakis I. I. (2006). Bone Remodeling. Ann. N. Y. Acad. Sci. 1092, 385–396. 10.1196/annals.1365.035, PMID: 17308163

[ref22] Hefferon K. L. Fan Y. (2004). Expression of a vaccine protein in a plant cell line using a geminivirus-based replicon system. Vaccine 23, 404–410. 10.1016/j.vaccine.2004.04.038, PMID: 15530687

[ref23] Hejnaes K. R. Ransohoff T. C. (2018). “Chemistry, manufacture and control,” in Biopharmaceutical Processing. eds. G. Jagschies, E. Lindskog, K. Łącki and P. Galliher, 1105–1136.

[ref24] Khan K. H. (2013). Gene expression in mammalian cells and its applications. Adv. Pharm. Bull. 3, 257–263. 10.5681/apb.2013.042, PMID: 24312845 PMC3848218

[ref25] Kleizen B. Braakman I. (2004). Protein folding and quality control in the endoplasmic reticulum. Curr. Opin. Cell Biol. 16, 343–349. 10.1016/j.ceb.2004.06.012, PMID: 15261665

[ref26] Kostenuik P. J. Nguyen H. Q. McCabe J. Warmington K. S. Kurahara C. Sun N.. (2009). Denosumab, a fully human monoclonal antibody to RANKL, inhibits bone resorption and increases BMD in knock-in mice that express chimeric (murine/human) RANKL. J. Bone Miner. Res. 24, 182–195. 10.1359/jbmr.081112, PMID: 19016581

[ref27] Leuzinger K. Dent M. Hurtado J. Stahnke J. Lai H. Zhou X.. (2013). Efficient agroinfiltration of plants for high-level transient expression of recombinant proteins. J. Vis. Exp. 77:50521. 10.3791/50521, PMID: 23913006 PMC3846102

[ref28] Lewiecki E. M. (2010a). Bisphosphonates for the treatment of osteoporosis: insights for clinicians. Ther. Adv. Chronic Dis. 1, 115–128. 10.1177/2040622310374783, PMID: 23251734 PMC3513863

[ref29] Lewiecki E. M. (2010b). Treatment of osteoporosis with denosumab. Maturitas 66, 182–186. 10.1016/j.maturitas.2010.02.008 20236778

[ref30] Makras P. Delaroudis S. Anastasilakis A. D. (2015). Novel therapies for osteoporosis. Metabolism 64, 1199–1214. 10.1016/j.metabol.2015.07.011, PMID: 26277199

[ref31] Montero-Morales L. Steinkellner H. (2018). Advanced plant-based glycan engineering. Front. Bioeng. Biotechnol. 6:81. 10.3389/fbioe.2018.00081, PMID: 29963553 PMC6010556

[ref32] NIH Consensus Development Panel on Osteoporosis Prevention and Therapy (2001). Osteoporosis prevention, diagnosis, and therapy. JAMA 285, 785–795. 10.1001/jama.285.6.785, PMID: 11176917

[ref33] Ono T. Hayashi M. Sasaki F. Nakashima T. (2020). RANKL biology: bone metabolism, the immune system, and beyond. Inflamm. Regen. 40:2. 10.1186/s41232-019-0111-3, PMID: 32047573 PMC7006158

[ref34] Petruccelli S. Otegui M. S. Lareu F. Tran Dinh O. Fitchette A.-C. Circosta A.. (2006). A KDEL-tagged monoclonal antibody is efficiently retained in the endoplasmic reticulum in leaves, but is both partially secreted and sorted to protein storage vacuoles in seeds. Plant Biotechnol. J. 4, 511–527. 10.1111/j.1467-7652.2006.00200.x, PMID: 17309727

[ref35] Phoolcharoen W. Bhoo S. H. Lai H. Ma J. Arntzen C. J. Chen Q.. (2011). Expression of an immunogenic Ebola immune complex in *Nicotiana benthamiana*. Plant Biotechnol. J. 9, 807–816. 10.1111/j.1467-7652.2011.00593.x, PMID: 21281425 PMC4022790

[ref36] Pillet S. Couillard J. Trépanier S. Poulin J. F. Yassine-Diab B. Guy B.. (2019). Immunogenicity and safety of a quadrivalent plant-derived virus like particle influenza vaccine candidate-two randomized phase II clinical trials in 18 to 49 and ≥50 years old adults. PLoS One 14:e216533. 10.1371/journal.pone.0216533, PMID: 31166987 PMC6550445

[ref37] Pogue G. P. Vojdani F. Palmer K. E. Hiatt E. Hume S. Phelps J.. (2010). Production of pharmaceutical-grade recombinant aprotinin and a monoclonal antibody product using plant-based transient expression systems. Plant Biotechnol. J. 8, 638–654. 10.1111/j.1467-7652.2009.00495.x, PMID: 20514694

[ref38] Rattanapisit K. Abdulheem S. Chaikeawkaew D. Kubera A. Mason H. S. Ma J. K.. (2017). Recombinant human osteopontin expressed in Nicotiana benthamiana stimulates osteogenesis related genes in human periodontal ligament cells. Sci. Rep. 7:17358. 10.1038/s41598-017-17666-7, PMID: 29229947 PMC5725595

[ref39] Rattanapisit K. Shanmugaraj B. Manopwisedjaroen S. Purwono P. B. Siriwattananon K. Khorattanakulchai N.. (2020). Rapid production of SARS-CoV-2 receptor binding domain (RBD) and spike specific monoclonal antibody CR3022 in *Nicotiana benthamiana*. Sci. Rep. 10:17698. 10.1038/s41598-020-74904-1, PMID: 33077899 PMC7573609

[ref40] Robblee J. Collins B. E. Kaundinya G. Bosques C. J. (2017). Methods Related To Denosumab. U.S. Patent Application.

[ref41] Saint-Jore-Dupas C. Faye L. Gomord V. (2007). From planta to pharma with glycosylation in the toolbox. Trends Biotechnol. 25, 317–323. 10.1016/j.tibtech.2007.04.008, PMID: 17493697

[ref42] Schähs M. Strasser R. Stadlmann J. Kunert R. Rademacher T. Steinkellner H. (2007). Production of a monoclonal antibody in plants with a humanized N-glycosylation pattern. Plant Biotechnol. J. 5, 657–663. 10.1111/j.1467-7652.2007.00273.x, PMID: 17678502

[ref43] Schoberer J. Strasser R. (2018). Plant glyco-biotechnology. Semin. Cell Dev. Biol. 80, 133–141. 10.1016/j.semcdb.2017.07.005, PMID: 28688929

[ref44] Shanmugaraj B. Bulaon C. J. I. Phoolcharoen W. (2020a). Plant molecular farming: a viable platform for recombinant biopharmaceutical production. Plan. Theory 9:842. 10.3390/plants9070842, PMID: 32635427 PMC7411908

[ref45] Shanmugaraj B. Phoolcharoen W. (2021). Addressing demand for recombinant biopharmaceuticals in the COVID-19 era. Asian Pac. J. Trop. Med. 14, 49–51. 10.4103/1995-7645.306736

[ref46] Shanmugaraj B. Rattanapisit K. Manopwisedjaroen S. Thitithanyanont A. Phoolcharoen W. (2020b). Monoclonal antibodies B38 and H4 produced in *Nicotiana benthamiana* neutralize SARS-CoV-2 *in vitro*. Front. Plant Sci. 11:589995. 10.3389/fpls.2020.589995, PMID: 33329653 PMC7728718

[ref47] Sheludko Y. V. Sindarovska Y. R. Gerasymenko I. M. Bannikova M. A. Kuchuk N. V. (2007). Comparison of several Nicotiana species as hosts for high-scale agrobacterium-mediated transient expression. Biotechnol. Bioeng. 96, 608–614. 10.1002/bit.21075, PMID: 16983697

[ref48] Sözen T. Özışık L. Başaran N. Ç. (2017). An overview and management of osteoporosis. Eur. J. Rheumatol. 4, 46–56. 10.5152/eurjrheum.2016.048, PMID: 28293453 PMC5335887

[ref49] Stelter S. Paul M. J. Teh A. Y. H. Grandits M. Altmann F. Vanier J.. (2020). Engineering the interactions between a plant-produced HIV antibody and human Fc receptors. Plant Biotechnol. J. 18, 402–414. 10.1111/pbi.13207, PMID: 31301102 PMC6953194

[ref50] Strasser R. (2016). Plant protein glycosylation. Glycobiology 26, 926–939. 10.1093/glycob/cww023, PMID: 26911286 PMC5045529

[ref51] Strasser R. Stadlmann J. Schahs M. Stiegler G. Quendler H. Mach L.. (2008). Generation of glyco-engineered *Nicotiana benthamiana* for the production of monoclonal antibodies with a homogeneous human-like N-glycan structure. Plant Biotechnol. J. 6, 392–402. 10.1111/j.1467-7652.2008.00330.x, PMID: 18346095

[ref52] Tremouillaux-Guiller J. Moustafa K. Hefferon K. Gaobotse G. Makhzoum A. (2020). Plant-made HIV vaccines and potential candidates. Curr. Opin. Biotechnol. 61, 209–216. 10.1016/j.copbio.2020.01.004, PMID: 32058899

[ref53] Tu K. N. Lie J. D. Wan C. K. V. Cameron M. Austel A. G. Nguyen J. K.. (2018). Osteoporosis: a review of treatment options. Phys. Ther. 43, 92–104., PMID: 29386866 PMC5768298

[ref54] Tusé D. Tu T. McDonald K. A. (2014). Manufacturing economics of plant-made biologics: case studies in therapeutic and industrial enzymes. Biomed. Res. Int. 2014:256135. 10.1155/2014/256135, PMID: 24977145 PMC4058100

[ref55] Uversky V. N. (2013). “Posttranslational Modification,” in Brenner’s Encyclopedia of Genetics. eds. S. Maloy and K. Hughes, 425–430.

[ref56] Veen S. J. Hollak C. E. M. Kuilenburg A. B. P. Langeveld M. (2020). Developments in the treatment of Fabry disease. J. Inherit. Metab. Dis. 43, 908–921. 10.1002/jimd.12228, PMID: 32083331 PMC7540041

[ref57] Wang T. T. Ravetch J. V. (2019). Functional diversification of IgGs through Fc glycosylation. J. Clin. Invest. 129, 3492–3498. 10.1172/JCI130029, PMID: 31478910 PMC6715372

[ref58] Wu X. Li Z. Yang Z. Zheng C. Jing J. Chen Y.. (2012). Caffeic acid 3,4-dihydroxy-phenethyl ester suppresses receptor activator of NF-kappaB ligand-induced osteoclastogenesis and prevents ovariectomy-induced bone loss through inhibition of mitogen-activated protein kinase/activator protein 1 and Ca^2+^-nuclear factor of activated T-cells cytoplasmic 1 signaling pathways. J. Bone Miner. Res. 27, 1298–1308. 10.1002/jbmr.1576, PMID: 22337253

[ref59] Xu J. Dolan M. C. Medrano G. Cramer C. L. Weathers P. J. (2012). Green factory: plants as bioproduction platforms for recombinant proteins. Biotechnol. Adv. 30, 1171–1184. 10.1016/j.biotechadv.2011.08.020, PMID: 21924345

[ref60] Xu J. Ge X. Dolan M. C. (2011). Towards high-yield production of pharmaceutical proteins with plant cell suspension cultures. Biotechnol. Adv. 29, 278–299. 10.1016/j.biotechadv.2011.01.002, PMID: 21236330

[ref61] Yin J. Li G. Ren X. Herrler G. (2007). Select what you need: a comparative evaluation of the advantages and limitations of frequently used expression systems for foreign genes. J. Biotechnol. 127, 335–347. 10.1016/j.jbiotec.2006.07.012, PMID: 16959350

[ref62] Zaheer S. Leboff M. Lewiecki E. M. (2015). Denosumab for the treatment of osteoporosis. Expert Opin. Drug Metab. Toxicol. 11, 461–470. 10.1517/17425255.2015.1000860, PMID: 25614274 PMC4480604

[ref63] Zhang B. Shanmugaraj B. Daniell H. (2017). Expression and functional evaluation of biopharmaceuticals made in plant chloroplasts. Curr. Opin. Chem. Biol. 38, 17–23. 10.1016/j.cbpa.2017.02.007, PMID: 28229907 PMC5767337

