# Supplementary material for: Expression and Functional Evaluation of Recombinant Anti-receptor Activator of Nuclear Factor Kappa-B Ligand Monoclonal Antibody Produced in Nicotiana benthamiana
Source: Front Plant Sci. 2021 Jun 23;12:683417. doi: 10.3389/fpls.2021.683417 (PMC8261044; doi:10.3389/fpls.2021.683417)
Supplement: Supplementary file 1 [file Data_Sheet_1.docx]

**Expression and Functional Evaluation of Recombinant Anti-receptor Activator of Nuclear Factor Kappa-B Ligand Monoclonal Antibody Produced in**

***Nicotiana benthamiana***

Wanuttha Boonyayothin ^1,2^, Sirorut Sinnung ^3^, Balamurugan Shanmugaraj ^1,2^, Yoshito Abe ^4^, Richard Strasser ^5^, Prasit Pavasant ^3,6^ and Waranyoo Phoolcharoen ^1,2*^

^1^ Research Unit for Plant-Produced Pharmaceuticals, Chulalongkorn University, Bangkok, Thailand

^2^ Department of Pharmacognosy and Pharmaceutical Botany, Faculty of Pharmaceutical Sciences, Chulalongkorn University, Bangkok, Thailand

^3^ Center of Excellence in Regenerative Dentistry, Faculty of Dentistry, Chulalongkorn University, Bangkok, Thailand

^4^ Department of Pharmaceutical Sciences, School of Pharmacy at Fukuoka, International University of Health and Welfare, Okawa, Japan

^5^ Department of Applied Genetics and Cell Biology, University of Natural Resources and Life Sciences, Vienna, Austria

^6^ Department of Anatomy, Faculty of Dentistry, Chulalongkorn University, Bangkok, Thailand

Department of Anatomy, Faculty of Dentistry, Chulalongkorn University, Bangkok, Thailand

*** Correspondence:** Waranyoo.P@chula.ac.th; Tel: 662-218-8359; Fax: 662-218-8357

**Figure S1.** The comparison of amino acid sequences of anti-RANKL mAb from DrugBank (accession number: DB06643) and the commercially available denosumab (Robblee et al., 2017) are listed below.

HC:


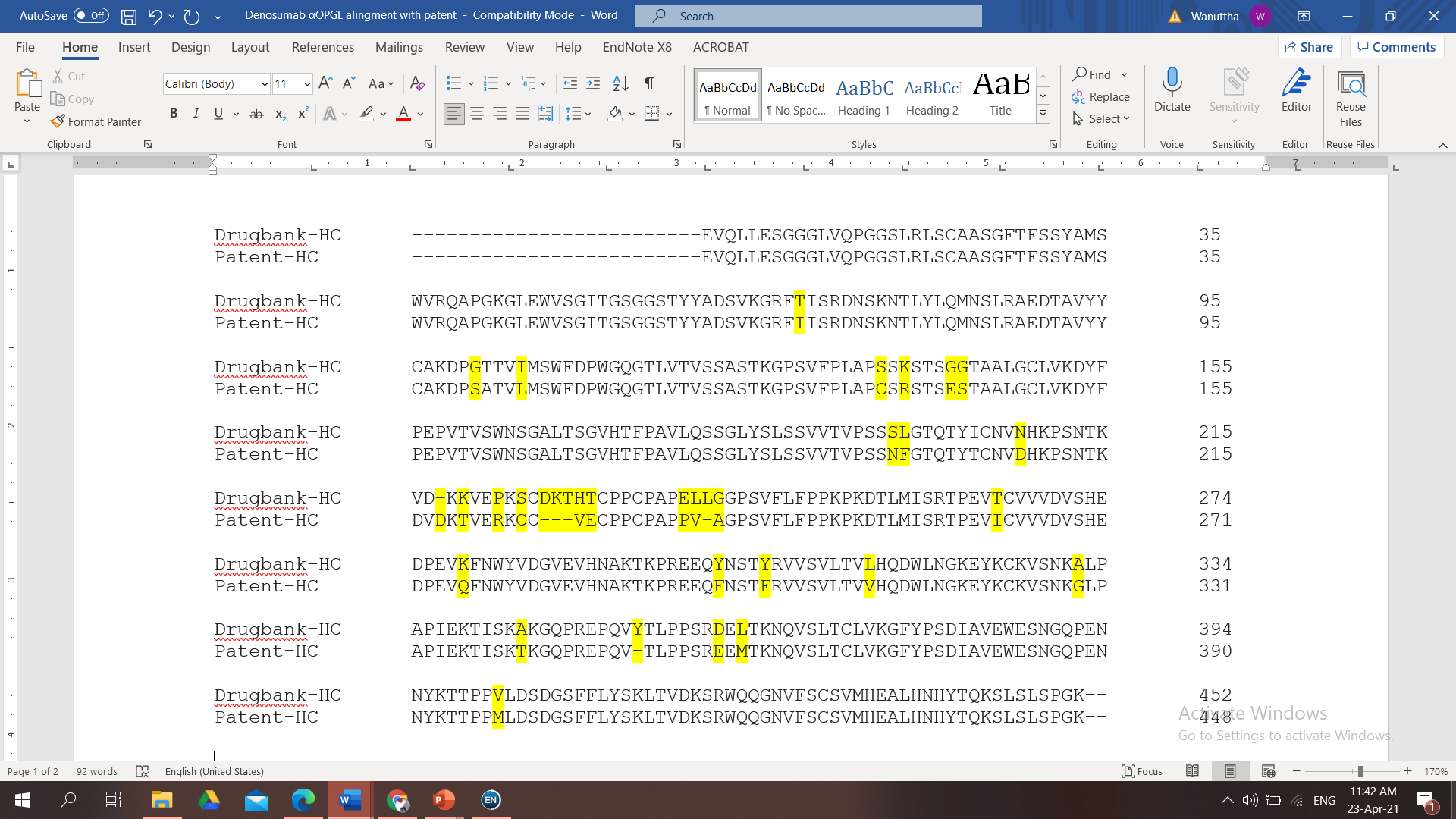


LC:


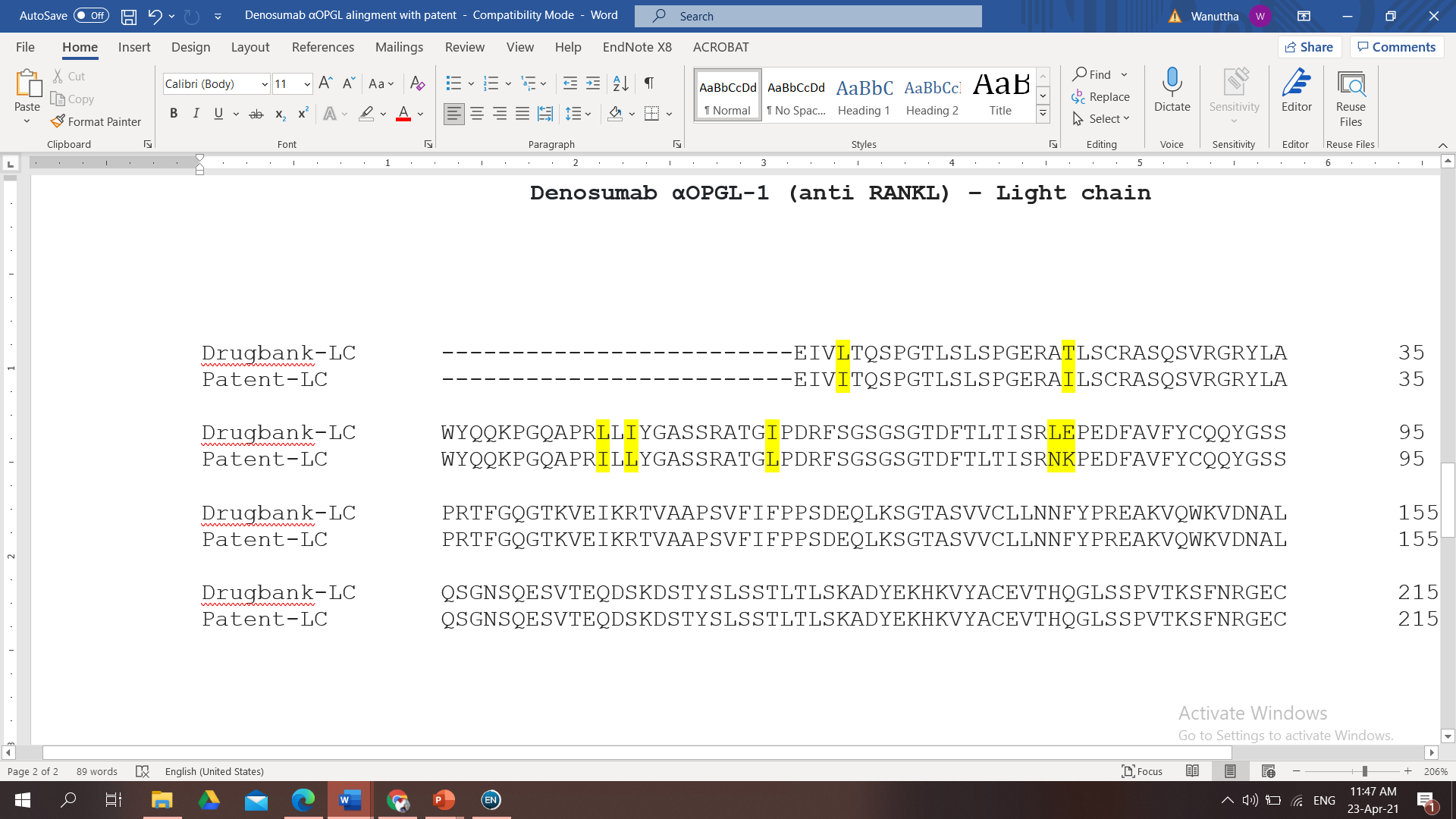


**Reference**

Robblee, J., Collins, B.E., Kaundinya, G., and Bosques, C.J. (2017). *Methods Related To Denosumab*. U.S. patent application.
